# Adenomyoepithelioma of the breast with late pulmonary metastases – case report and review of the literature

**DOI:** 10.1186/s13019-016-0518-8

**Published:** 2016-08-04

**Authors:** Agnieszka Korolczuk, Magdalena Amarowicz, Kamila Bąk, Elżbieta Korobowicz, Tomasz Koncewicz

**Affiliations:** 1 Department of Clinical Pathomorphology, Medical University, Jaczewskiego 8, 20-059 Lublin, Poland; 2 Chair and Department of Thoracic Surgery, Medical University, Lublin, Poland

**Keywords:** Adenomyoepithelioma, Breast, Epithelial-myoepithelial carcinoma, Lung

## Abstract

**Background:**

Adenomyoepithelioma (AME) of the breast is a rare tumour of unpredictable clinical behaviour. Most of the tumours are benign with some giving local recurrences or distant metastases.

**Case Presentation:**

We report a case of late lung metastases in a woman with a history of breast adenomyoepithelioma. Partial lobectomy was performed for lung lesions and initial diagnosis was epithelial-myoepithelial carcinoma.

**Conclusion:**

Careful slide’s revision of both breast and pulmonary lesions showed identical microscopic appearance with lung tumour performing more malignant features. Tumour cells in both: breast and pulmonary lesions were positive for cytokeratin and EMA (epithelial cells) and also for SMA, S100 and vimentin (myoepithelial cells). Two years and 7 months follow-up showed no recurrent neoplastic disease in our patient.

## Background

Adenomyoepithelioma (AME) is a tumour characterized by a bicellular proliferation consisting of glands with an inner epithelial and an outer myoepithelial cell layer. The classification of the World Health Organization [1] divides the adenomyoepithelioma into a benign type where both the epithelial and myoepithelial component are histologically non-malignant and a form, which shows a malignant transformation [2].

Adenomyoepithelioma can be found in salivary gland, skin adnexal, lung and this tumor may develop in breast [2–5]. It is generally considered to be benign or to show a low-grade malignancy. For the first time this tumour was reported in the breast by Hamperl in 1970 [6, 7]. The biological behavior of tumours developing in mammary glands may range from benign to malignant transformation of either epithelial or myoepithelial component separately or both [2, 7, 8]. The age of patients with breast adenomyoepithelioma ranges from 26 to 82 years, with an average of around 60 years [9]. These lesions develop usually as single foci with possible infiltration of surrounding breast tissue [8]. Foci of calcification may be seen on ultrasound examination. These cases that exhibit aspects of malignant transformation are rare in the literature. The myoepithelial cells express cytokeratins of the basal layer of stratified epithelia (CK5, CK14, and CK17), α-smooth muscle actin (SMA) and the heavy chain-myosin (hc-myosin). Some tumor suppressor proteins, including p63, p73, 14-3-3 sigma, maspin and Wilms Tumor (WT-1) have been preferentially detected in myoepithelial cells [2]. Morphological features of malignancy that could predict the potential for local recurrence and/or metastasis are not well-established. Cellular pleomorphism, mitoses, necrosis, invasion of the surrounding tissue and association with other types of malignant tumors such as invasive ductal carcinoma and undifferentiated carcinoma are thought to be the most important [10].

Epithelial-myoepithelial carcinoma occurs most frequently in both major and minor salivary glands, where accounts for approximately 1 % of primary neoplasms. It belongs to low-grade tumours that may locally recur and less frequently metastasise. Other known locations for these tumours are skin and breast [11]. Primary epithelial-myoepithelial carcinoma of lung has only recently been described [12–15]. Myoepithelial cells are believed to play an important role in the development of this type of tumours. Subcellular aberrant location of p27/kip-1 seems to be crucial in loss of growth-inhibition function and uncontrolled proliferation of myoepithelial cells [11]. In most of these tumours no aggressive clinical course has been noted, though in the recently described cases the follow-up time has been too short for the assessment of their clinical behaviour.

The aim of the study is to present a case of late pulmonary metastases of breast adenomyoepithelioma in 57 years old woman.

## Case Presentation

**Fig. 1:**
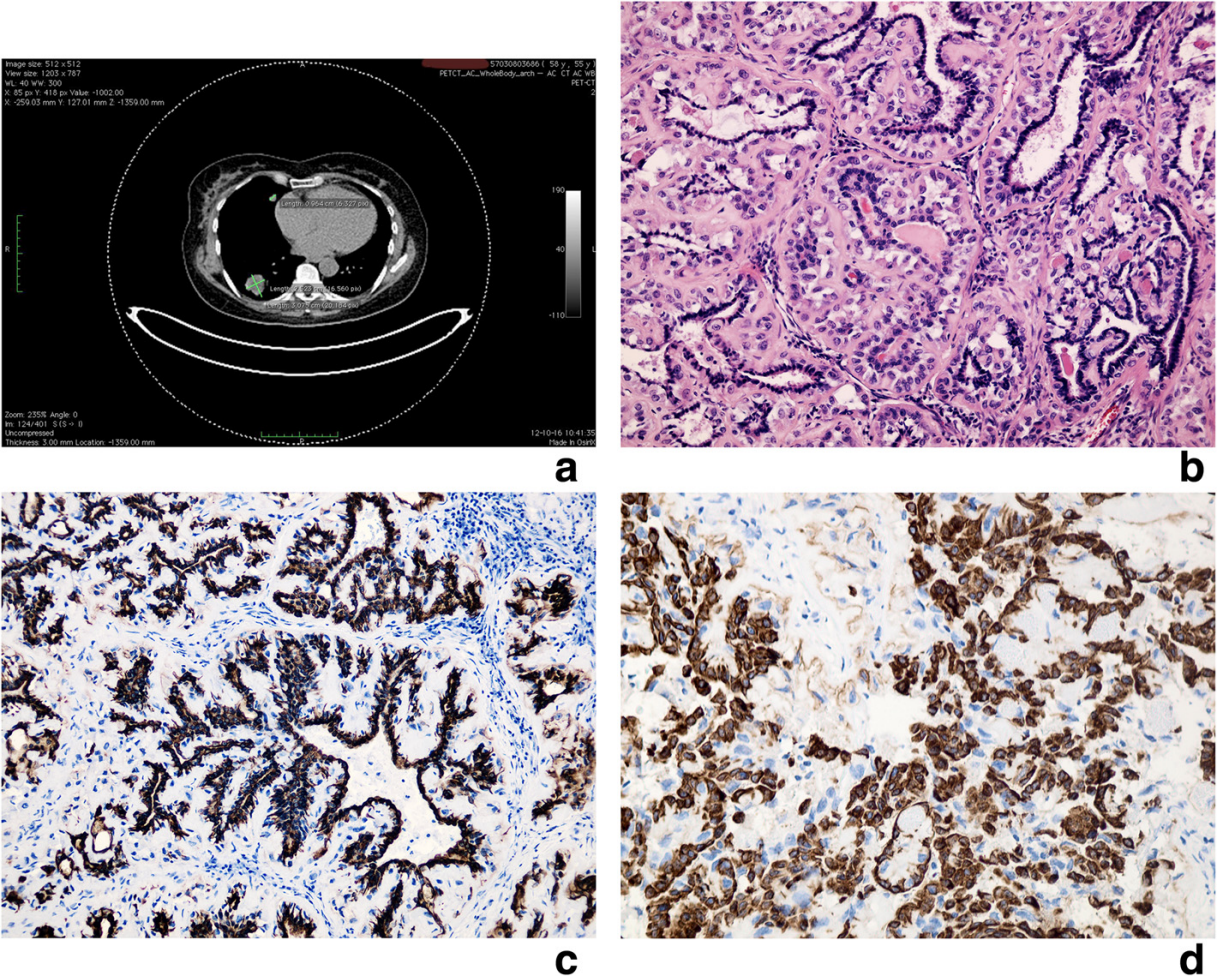
Lung tumour: Metastatic adenomyoepithelioma with component of epithelial-myoepithelial carcinoma. **a** PET-CT scan: Two nodular masses located in right lower (measuring 26x31mm) and middle lobe (diameter 96 mm). **b** Light microscope. Inner layer of glandular structures lined by epithelial cells, outer layer formed by myoepithelial cells with clear cytoplasm, mild nuclear atypia and some nucleoli visible. Homogenous, hyalinised stroma. H + E x400. **c** Immunochistochemical staining for cytokeratin positive in epithelial cells. x400. **d** Immunochistochemical staining for SMA positive in myoepithelial cells. x400

Fifty six years old woman had a history of breast tumour and had left-side mastectomy in 2007. The control chest radiograph and computed tomography (CT) scan performed in 2012 revealed two nodular masses located in her right lower (measuring 26x31mm) and middle lobe (diameter 96 mm) (Fig. 1a). No associated enlarged lymph nodes were found. The patient had no history of smoking or pulmonary infectious disease. She underwent positron emission tomography-computed tomography (PET-CT) scan that showed low-grade uptake with an SUV of max 6,2 after 120 min within both pulmonary lesions. There was no uptake within other parts of the body, including breast and salivary glands. Therefore, a pulmonary lower and middle sleeve lobectomy was performed and the material was obtained for histopathological examination.

## Material and methods

Formalin fixed and paraffin-embedded sections were stained with hematoxylin and eosin (H + E) and Periodic acid Schiff (PAS). Additional sections were stained with panel of antibodies listen in Table 1. A commercially available detection kit (Dako Envision Plus-HRP, DAKO, Glostrup, Denmark) was used according to the manufacture’s instruction. Slides were lightly counterstained with 1 % Harris’ hematoxylin.

**Table 1:** Reagents used in immunohistochemical characterization for breast and pulmonary lesions

Antibody	Dilution	Source	Result: epithelial cells	Results: myoepithelial cells
Cytokeratin pool	1:100	DAKO	positive	negative
S-100 protein	1:3000	DAKO	negative	positive
Smooth muscle actin	1:50	DAKO	negative	positive
p63 protein	1:100	ZECA Corp	negative	negative
TTF-1	1:50	DAKO	negative	negative
Ki-67 antigen^a^	1:50	DAKO	Positive	positive
EMA	1:50	DAKO	Positive	negative
Vimentin	1:50	DAKO	negative	positive
PR	1:50	DAKO	negative	negative
ER	1:50	DAKO	negative	negative

^a^Ki-67 positive in 4,5 % of tumour cells (epithelial and myoepithelial cells) in

pulmonary lesions. Not performed on breast tumour

As breast lesion was diagnosed in different laboratory (in 2007), we have asked for and reviewed the slides and immunohistochemical stainings. The lesion at that time was diagnosed as adenomyoepithelioma and two separate laboratories confirmed the diagnosis. We have made the revision of breast tumour slides as well as immunohistochemical stainings.

## Results

Gross examination of the right lung lower lobe revealed a circumscribed, nonencapsulated nodular mass measuring 35x30x30mm infiltrating focally the bronchial wall (the bronchus intermedius). The cross section was uniform and grey with punctate foci of necrosis. Examination of middle lobe revealed well circumscribed nodule measuring 10x7mm located close to the bronchial wall, with no infiltration nor necrosis grossly visible.

Microscopic examination of revealed in both lesions unencapsulated, polypoid epithelial neoplasms that infiltrated focally the bronchial wall and pulmonary parenchyma. The tumour mass was composed of two types of cells that formed glandular or duct-like pattern (Fig. 1b). The inner (luminal) layer of cells had eosinophilic cytoplasm and centrally located uniform nuclei without nucleoli. Large polygonal cells with clear cytoplasm and uniform nuclei formed the outer layer (Fig. 1b). The duct-like structures contained PAS positive material in the luminal spaces. The stroma had hyalinised and eosinophilic appearance (Fig. 1b). Mitotic figures were present (<5 mitotic figures/10 high power fields-HPF). Immunohistochemical studies confirmed the biphasic nature of the tumour (Fig. 1c, d). Results are summarized in Table 1. There were microscopic focal necrosis, nuclear atypia and signs of the adjacent pulmonary parenchyma infiltration. No lymphovascular space invasion was noticed. The initial diagnosis was epithelial-myoepithelial carcinoma.

**Fig. 2:**
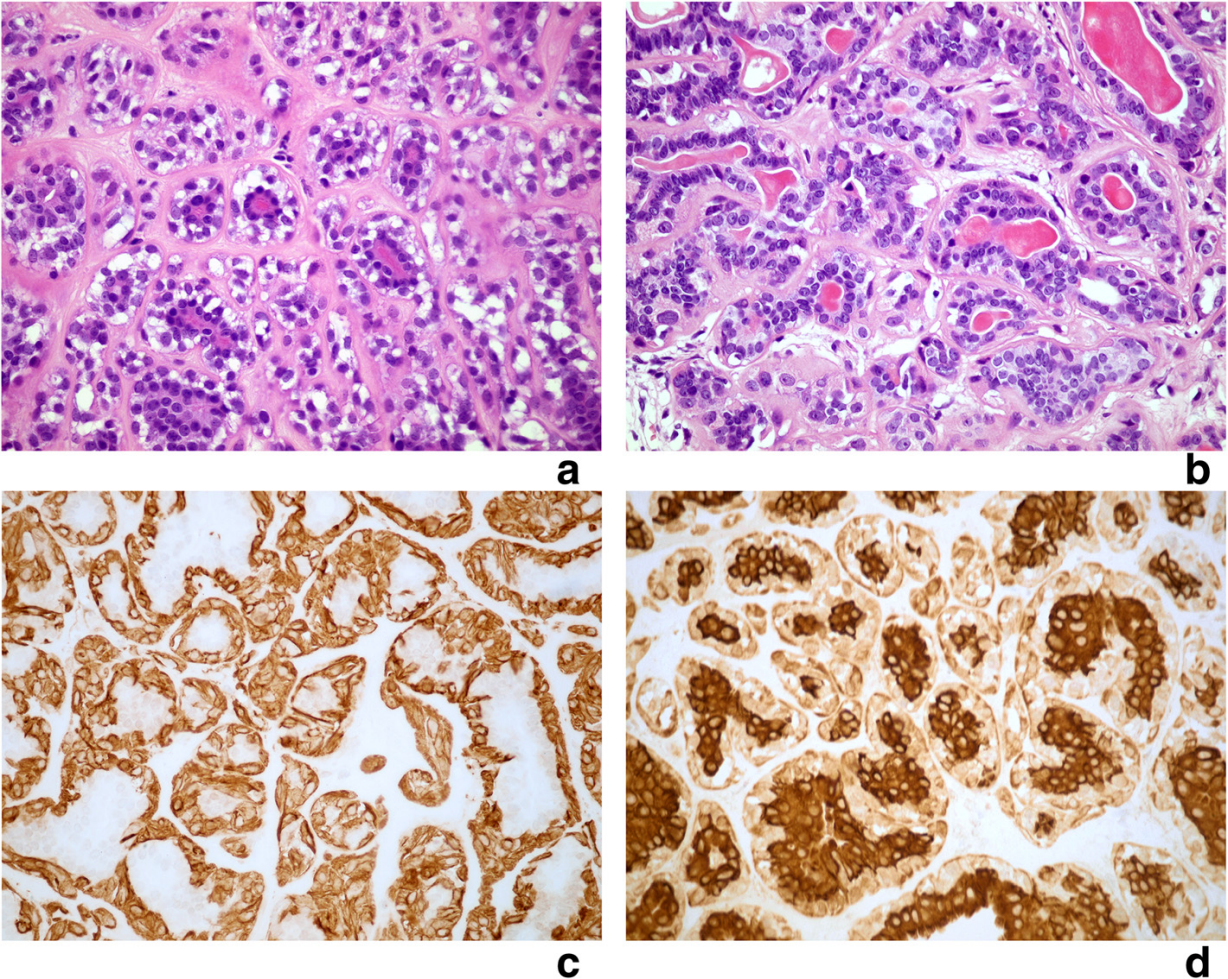
Breast tumour: Adenomyoepithelioma. **a**, **b** Light microscope. Inner layer of glandular structures lined by epithelial cells, outer layer formed by myoepithelial cells with clear cytoplasm, mild nuclear atypia and single mitotic figures. Homogenous, hyalinised stroma. H + E x400. **c** Immunochistochemical staining for SMA positive in myoepithelial cells. x400. **d** Immunochistochemical staining for cytokeratin positive in epithelial cells. x400

Gross description that we found in the diagnosis while revising microscopic slides of the breast lesions revealed the information about two separate firm, well demarcated lesions within the left breast measuring: 26x18x16mm and 22x20x18mm respectively. Microscopic appearance of revised slides was consistent with the diagnosis of adenomyoepithelioma (Fig. 2a, b). However single mitotic figures were present (Fig. 2b) Immunohistochemical study showed identical immunohistochemical profile with above presented lung lesion (Table 1, Fig. 2c, d). The hormone estrogen and progesterone receptors were negative in both: breast and lung tumours.

## Discussion

Adenomyoepithelioma of the breast is a rare disorder characterized by the simultaneous proliferation of ductal epithelium and myoepithelial cells. In 1991, Tavassoli [9] classified these tumours according to their malignant potential and subdivided them into benign and malignant lesions in which one of/or both components can perform malignant features. In 2003, this classification was adopted by WHO. The average age of onset is around 60 years [3, 9, 16] (our patient was at 50 years when breast tumour was diagnosed). Patients usually present a solitary, well-circumscribed, sometimes palpable nodule (depending on location and size), measuring on average 20–50 mm [3, 17].

No risk factors are reported for this type of tumours [16, 18]. The mammographic and sonographic signs are not specific; however they can guide the diagnosis and give a precise assessment of the lesions [16]. The surgical removal is the treatment of choice for both benign and malignant tumours [3, 17]. Local recurrence has been described and is more common if the margin of excision is narrow or there is incomplete excision [3].

Microscopic examination reveals formation of papillary or micropapillary, tubular, cystic or solid areas formed by neoplastic cells. Single tumour may represent a mixture of these components, or one of the components may replace most of tumour mass [3, 16–19]. In some cases the solid areas were prominent [3, 4, 17]. PAS positive, diastase-sensitive amorphous material is seen within the tubular lumina or intercellular spaces. In some cases the stroma is hyalinised or shows myxoid change. In some cases focal atypia, necrosis, and presence of mitotic figures were reported and are usually associated with cases that either recurred or had a malignant clinical outcome [3, 4, 17, 20].

The review of breast tumour slides in our case revealed mostly tubular microscopic appearance with hyalinised stroma. Single mitotic figures and focal mild nuclear atypia were present (Fig. 2a, b).

The cytoplasm of epithelial cells of AME uniformly reacts with antibodies to cytokeratins, such as cytokeratin AE1/3 (CAM 5.2, or CK7.6, 7) [2–4, 18]. The luminal surfaces of the glandular cells are positive for the epithelial membrane antigen. The myoepithelial component reacts with p63, smooth muscle myosin (SMA) heavy chains, CK5, CD10, calponin, actin, and S100 [2, 3, 7, 16, 18]. Proliferative index of Ki-67 immunostaining is present in both compartments of the tumor but may be higher in the myoepithelial cells than it is in the ductal cells [3, 17, 21]. Immunostaining for estrogen is either negative or weakly positive in a patchy pattern. Progesterone receptor and ERBB2 (formerly Her2/neu) have, however, been consistently reported to be negative in all the published studies [3, 22].

Immunohistochemical results of the breast lesion diagnosed in our patient are consistent with desribed above and presented in Table 1.

Differential diagnosis should include intraductal papilloma, intraductal hyperplasia, ductal carcinoma or nipple adenoma. A diagnosis of AME is favored if myoepithelial proliferation is extensive and involves the lesion diffusely [3, 5, 18]. In nipple adenoma presence of florid ductal hyperplasia and the pseudoinfiltrative pattern without fibrous hyalinised stroma are helpfull features for differentation. Other tumours that should be included in the differential diagnosis are: fibroadenoma, phyllodes tumor or tubular adenoma with AME-like areas, ductal adenoma, and nodular adenosis, clear cell carcinoma, microglandular adenosis [3, 5, 18]. Microglandular adenosis is characterized by an absence of myoepithelial layer and S100 positivity [18].

The prognosis of benign and locally recurrent disease is good [3, 5, 17]. Chemotherapy has been used in few malignant cases without much success, however eribulin was recently presented by Lee et al. [5] to have a benefit effect on breast AME that presented distant metastases. Metastases to axillary lymph nodes may be reported [2–4, 17, 23]. Several cases with distant metastases have been reported, mainly to lungs [2, 5, 17], brain, thyroid, chest wall or even abdominal cavity [5, 23]. The prognosis of metastatic malignant adenomyoepithelioma is poor [5]. Epithelial-myoepithelial carcinoma (EMC), also reported in the breast, is extremely rare tumour in this location performing prominent malignant microscopic features such us: more solid structure, nuclear pleomorphism, high mitotic activity, areas of necrosis and infiltration of surrounding structures [21]. Moushine et al. [16] and Shah et al. [24] presented cases of EMC with described above malignant histologic features, arising in breast adenomyoepithelioma.

Our case presents a patient with lung tumours developing 5 years after breast adenomyoepithelioma. Microscopic appearance of lesions located in breast and lung was similar with lung tumours performing more malignant appearance: nuclear pleomorphism, mitotic figures, and focal necrosis. Immunohistochemical profile was identical in both: breast and lung tumours and is summarized in Table 1.

Such late presentation of metastases is unusual for the breast lesions of AMEs group. Nadelman [25] presented two cases with lung metastatic disease that were diagnosed at the same time and one year after breast tumour was diagnosed. Maffini [26] presented a case of malignant breast AME with lung metastasis one year later and Lee [5] reported metastatic lung tumour 10 months after the initial diagnosis of breast AME.

Primary pulmonary neoplasms showing a mixture of epithelial and myoepithelial elements are extremely infrequent with only 25 cases published, classified as “adenomyoepithelioma”, “pneumocytic adenomyoepithelioma”, “myoepithelioma” and epithelial-myoepithelial carcinoma”. The last known case about pulmonary neoplasm with biphasic morphology was published by Arif et in 2012 [27]. Epithelial-myopepithelial carcinoma is an uncommon, low-grade malignant salivary gland neoplasm characterized by neoplastic proliferation of epithelial and myoepithelial cells. Neoplasms with similar morphology have been reported in the breast, skin and lacrimal glands. This rare tumour has only been diagnosed in last two decades [11, 15, 28]. According to WHO this tumour is treated as malignant neoplasm composed of variable proportions of two types of cells, which typically form gland-like structures. The biphasic morphology is represented by an inner epithelial cells layer, enveloped by an outer layer of clear myoepithelial cells.

Pulmonary epithelial-myoepithelial carcinoma appears mainly in middle-aged patients (34–76 age range) with slight female predominance [11, 27]. The youngest reported patient was 7 years old boy [29]. Symptoms varied from asymptomatic cases to cough, hemoptysis, thoracic pain, fever, dyspnoea, pneumonia or recurrent infections [11, 30, 31]. The size of the tumour ranges from 0,8 to 16 cm (average 3,2 cm). Grossly, most of these neoplasms arise as well-defined, endobronchial, nonencapsulated polypoid mass [11]. In most described cases a connection to the wall of a bronchus was evident, less commonly the tumour presented as intraparenchymatous mass without apparent bronchial connection [32].

Immunohistochemically the inner epithelial cells layer shows positivity for EMA and cytokeratins: low-molecular weight but is negative for S-100 protein, vimentin and smooth muscle actin (SMA). The opposite reaction is present in outer myoepithelial cells layer; they are negative for EMA and cytokeratins but positive for S-100 protein, SMA and weekly for vimentin. Immunopositivity for calponin, CD117 and GFAP was reported in these cells [27]. These cells show positivity for p63, which is known as a marker of squamous epithelium. P63 positivity is restricted to basal cells of respiratory epithelium and peripheral cells of bronchial mucous glands that are believed to be the counterparts of myoepithelial cells. These observations suggest that p63 might be one of the markers of myoepithelial cells [30]. Ki-67 labeling is reported in few cases and it ranges from < 1 % to < 2 %.

Microscopic appearance of both lung tumours in our patient was consistent with above described pulmonary EMC. Pathologist, at the time of the diagnosis, was not aware of the type of primary breast lesion. The immunoprofile in both lesions was consistent with other cases of pulmonary epithelial-myoepithelial carcinoma reported, results are summarized in Table 1. Ki-67 was positive in approximately 4,5 % of tumour cells (epithelial and myoepithelial). TTF-1 was negative in both: epithelial and myoepithelial cells.

As the breast tumour in our patients that occurred 5 years prior to lung lesions have been diagnosed as adenomyoepithelioma, after the careful revision of breast tumour slides and patients history, we conclude that the lung tumours diagnosed as epithelial-myoepithelial carcinoma should be treated as late metastases of the breast malignant adenomyoepithelioma with metastatic tumour being more malignant. Presence of mitotic figures, signs of nuclear atypia, focal infiltration of pulmonary parenchyma as well as focal necrosis should be treated as component of epithelial-myoepithelial carcinoma. Similar microscopic appearance with primary breast tumour, identical immunohistochemical profile and two foci located within the same lung support metastatic rather that primary origin of pulmonary lesions. Several authors conclude that breast AMEs performing as tumours over 2 cm should be treated as potentially malignant [3, 5, 7, 8, 17]. Two primary breast lesions in our case were over 2 cm each. Histology of these tumours presented potential features of malignant behaviour (single mitotic figures, signs of nuclear atypia). However, for the best of our knowledge, such late presentation of distant metastases of breast AME as in our patient has never been reported before, and this makes the case unique.

## Conclusion

We conclude that atypical features should be noted in the pathology report of breast adenomyoepithelioma and careful follow-up should be employed, due to the lack of experience with these tumours, complete excision with adequate margins is recommended to decrease the potential for recurrence and metastasis. Precise criteria for microscopic diagnosis (benign versus malignant) as well as optimal treatment strategies remain to be determined.

The patient has not performed the further metastases since the lobectomy was done; she stays under the careful clinical and radiologic follow-up.

### Consent

Written informed consent was obtained from the patient for publication of this case report and any accompanying images. A copy of the written consent is available for review by the Editor-in-Chief of this journal.

## Abbreviations

AME, adenomyoepithelioma; CK, cytokeratin; CT, computed tomography; EMA, epithetlial membrane atigen; EMC, epithelial-myoepithelial carcinoma; ER, estrogen receptor; H + E, hematoxylin and eosin (H + E); PAS, periodic acid schiff; PET- CT, positron emission tomography-computed tomography; PR, progesterone receptor; SMA, smooth muscle actin; WHO, World Health Organisation
